# CT Images of a Severe TMJ Osteoarthritis and Differential Diagnosis with Other Joint Disorders

**DOI:** 10.1155/2013/242685

**Published:** 2013-12-05

**Authors:** K. L. Ferrazzo, L. B. Osório, V. A. Ferrazzo

**Affiliations:** ^1^School of Dentistry, Franciscan University Center, Andradas Street, 1614, 97010-032 Santa Maria, RS, Brazil; ^2^Department of Stomatology, School of Dentistry, Federal University of Santa Maria, Floriano Peixoto Street, 1184, 97015-372 Santa Maria, RS, Brazil

## Abstract

Osteoarthritis (OA) is the most common arthritis which affects the human body and can affect the temporomandibular joint (TMJ). The diagnosis of TMJ OA is essentially based on clinical examination. However, laboratory tests and radiographic exams are also useful to exclude other diseases. The diagnosis of OA may be difficult because of other TMJ pathologies that can have similar clinical and radiographic aspects. The purpose of this study was to describe an unusual case of bilateral TMJ OA in an advanced stage and discuss its most common clinical, laboratory, and radiographic findings, focusing on their importance in the differential diagnosis with other TMJ diseases. Erosion, sclerosis, osteophytes, flattening, subchondral cysts, and a reduced joint space were some of the radiographic findings in TMJ OA. We concluded that, for the correct differential diagnosis of TMJ OA, it is necessary to unite medical history, physical examination, laboratory tests, and radiographic findings. Computed tomography is the test of choice for evaluating bone involvement and for diagnosing and establishing the degree of the disease.

## 1. Introduction 

Osteoarthritis (OA) is a chronic noninflammatory degenerative condition that is the most common form of arthritis affecting the human body [1, 2]. Osteoarthrosis, deforming arthritis, and degenerative joint disease are the most used synonymous terms of OA [3]. In the pathogenesis of OA, evidence is growing for the role of systemic and biomechanical factors [2]. OA can be broadly divided into two groups: (1) primary osteoarthritis, when there is no previous pathology and the cause is unknown; (2) secondary osteoarthritis, when it is secondary to some previous injury, stress, or pathology in the joint [4–6]. The disease can be defined as a gradual loss of articular cartilage primarily, associated with thickening of the subchondral bone. The bone undergoes reactive hypertrophy forming peripheral osteophytes. Secondly, there is a mild, chronic nonspecific synovial inflammation. It most commonly affects middle-aged and older people with a predilection for women after the age of 50 [6]. Women with osteoarthritis of the hands often develop bony lumps at the ends of their fingers called Heberden's nodes. They most frequently occur in women over forty and may run in families. These nodes may be confined to one or several fingers. They are painless, grow gradually, and are not progressive [6, 7]. Although OA occurs more frequently in the joints of the hips, knees, and spine, which support more weight, it also affects the neck, hands, and temporomandibular joint (TMJ). In the TMJ, the most common signs and symptoms of OA are swelling and palpable tenderness of the joint, crepitation, and limited mandibular movement. Joint pain is usually mild in the morning and gets worse in the evening after a day's activity [1, 6–11].

The diagnosis of TMJ OA is mainly based on medical history and clinical examination. There are no specific laboratory tests to make a definitive diagnosis of OA. Results of laboratory tests such as rheumatoid factor (RF), antinuclear antibody (ANA), and erythrocyte sedimentation rate (ESR) are normal and are, therefore, useful only to rule out other diagnoses [7]. For complete analyses, imaging examinations are required. Panoramic and conventional radiographs may identify rough TMJ changes, but these methods are restricted in diagnosis, because of the anatomical superposition that prevents accurate view of the bone components. In this way, computed tomography (CT) is a useful exam that helps to confirm the diagnosis of TMJ OA and also to grade its severity [7, 8].

In the current paper, we present an unusual case of bilateral TMJ OA in an advanced stage focusing on clinical, laboratory, and radiographic differential diagnosis of the disease.

## 2. Case Report 

A 68-year-old white female presented with the main complaint of moderate pain in the TMJ (preauricular region), and a reduced opening of the mouth. Her medical history revealed good general health. Curiously, during anamnesis she reported she was involved in a car accident 10 years before and had a mandible injury, which caused only a chin laceration and local swelling. However, the patient associated the injury with the beginning of the symptoms—on occasion there was a severe bilateral pain in TMJ, with reduction on mandibular movements for about five weeks. At that time, she was treated with nonsteroidal anti-inflammatory drugs until the disappearance of the symptoms, when she recuperated the movement limitation.

**Figure 1:**
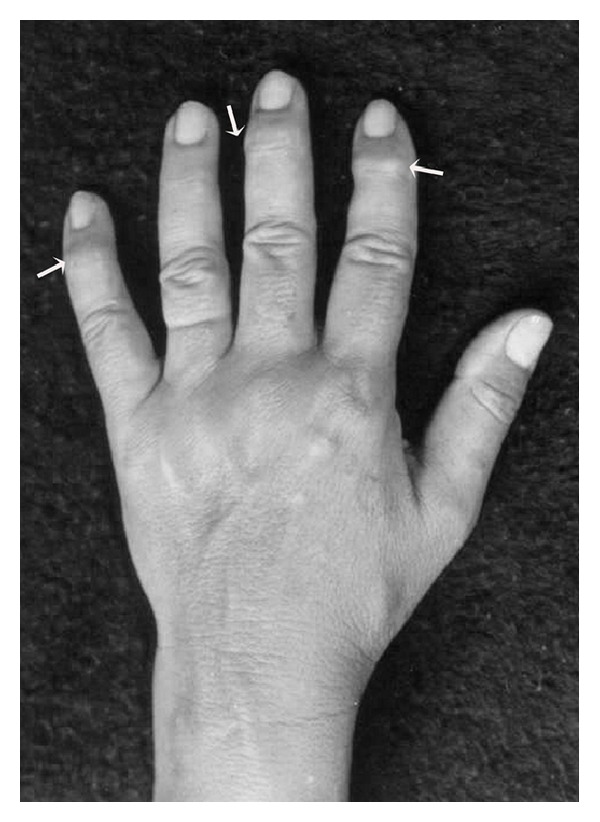
Bony growth spurs at the joint at the end of the fingers—Heberden's nodes (arrows).

At the present examination, intraoral investigation revealed a limitation of the vertical mouth opening (25 mm interincisally) and occlusion disorder with dental loss. The assessment of the other mandibular movements, such as lateral excursion or mandibular deflection, was not possible because of the pain and movements limitation. On physical examination, a characteristic enlargement at the distal interphalangeal joint, called Heberden's node (Figure 1), was seen. There was no familial history of arthrosis. Laboratory studies for evaluation were requested including complete blood count, erythrocyte sedimentation rate, rheumatoid factor, and antinuclear antibodies. All results were within normal limits.

**Figure 2:**
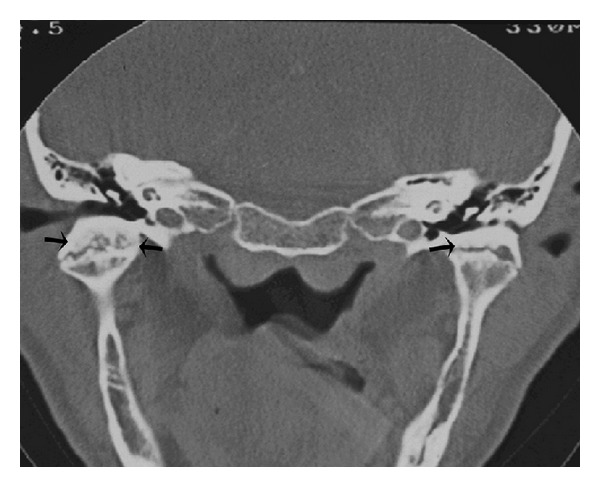
Coronal CT image demonstrating bilateral joint space narrowing, rough condylar surfaces, and sclerosis of the subchondral bone (arrows).

**Figure 3:**
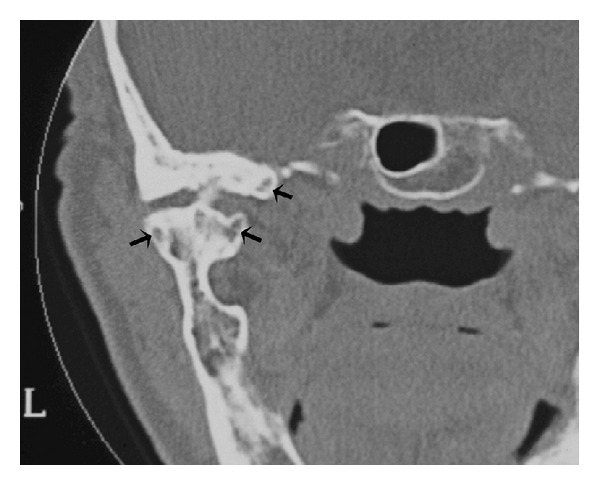
Subchondral cysts called Ely's cysts (arrows).

**Figure 4:**
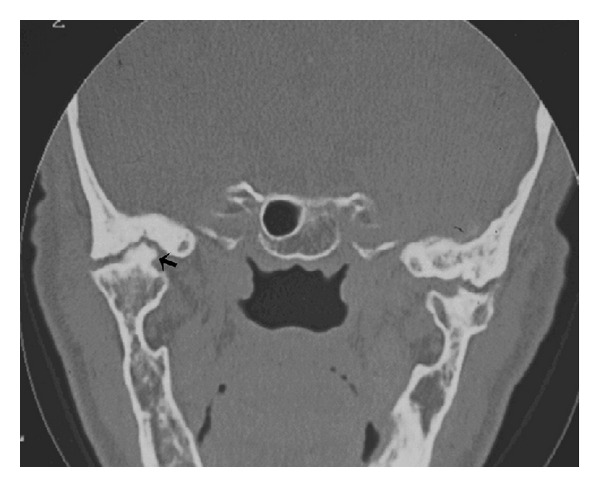
Large bone outgrowth (osteophyte) in the left TMJ (arrow) and bilateral subchondral cysts.

Computed tomography of the TMJ was performed, and coronal segments showed erosion of the articular surface of the condyle, rough condylar surfaces with bilateral joint space narrowing, thickening of the subchondral bone, sclerosis areas (Figure 2), subchondral cysts (Figure 3), and bone outgrowths—osteophytes (Figure 4). Although evaluation of disc position is important, it was not possible to take it as there was no magnetic resonance equipment at the public hospital, and also, the patient could not afford it at a private service.

On the basis of the clinical and tomographic findings and negative laboratory tests that excluded other articular diseases, final diagnosis was bilateral osteoarthritis of the TMJ.

Regarding the therapy, a nonsurgical treatment with load reduction in the TMJ by modifying the patient's diet (liquid diet initially and, after that, some soft food) was firstly proposed. Moreover, an analgesic with myorelaxing effect 3 times a day during two weeks (flupirtine maleate 100 mg—Katadolon, Asta Medica, Frankfurt, Germany) was prescribed in order to reduce joint pain. She was monitored for pain control for 1 month. The pain assessment tool was the verbal rating scale. She was asked to rate verbally the level of perceived pain by selecting the category that best described her pain: none, mild, moderate, or severe pain. After two weeks, TMJ pain on palpation and on movement had completely disappeared, but the vertical mouth opening had not been improved.

The second step would be the surgical treatment, because of the limitation of mouth opening. The patient was then informed about the indication of surgical treatment and prognosis. She was submitted to the clinical management, which temporarily relieved her pain, but refused any surgical procedure. Therefore, she was only treated for pain control until she was lost for follow-up.

## 3. Discussion 

There are various conditions which are similar to TMJ OA and must be taken into account in the differential diagnosis. In this paper, the diagnosis of osteoarthritis was in accordance with the Research Diagnostic Criteria for temporomandibular disorders, regarding the physical signs and pain symptomatology [14]. Many patients with symptoms in the TMJ are frequently misdiagnosed as having myofascial pain dysfunction syndrome, and it is essential to consider other pathologies of the joint, because some of these diseases have different treatment planning. Primarily, the differential diagnosis of OA of the TMJ should include the rheumatoid arthritis (RA) and its variants, pain dysfunction syndrome (PDS), and various forms of internal derangement (ID) [12, 15]. However, the major difficulty is to differentiate OA from early PDS and RA [3]. The main features to distinguish among them are listed on Table 1.

Osteoarthritis has been classified as primary when no precipitating cause is apparent, and as secondary when a related or preexisting condition may lead to its development [4, 5]. From this point of view, our clinical case is uncertain because the patient associated the beginning of the symptoms with a trauma (secondary OA). Despite this, the presence of Heberden's nodes showed that the OA was not localized. Although the relationship between acute joint trauma and development of posttraumatic OA remains poorly understood, it is clear that traumas increase the risk for later OA [2]. Both Heberden's (distal interphalangeal joint) and Bouchard's (proximal interphalangeal joint) deformities can be observed in the hand of rheumatoid patients, but the first usually is more frequent in OA [3, 13, 16]. Proximal interphalangeal and metacarpophalangeal involvement are more common in RA [3, 16]. OA of TMJ usually affects both mandibular condyle and articular eminence resulting in erosion, sclerosis, osteophytes, flattening, subchondral cysts, and a reduced joint space [11, 17–21]. Therefore, accurate image exams are important in detecting osseous and soft tissue changes [7, 8, 11, 20]. Several image techniques to TMJ examination have been described, as conventional tomography, magnetic resonance imaging, computed tomography, and, more recently, cone beam computed tomography [7, 8, 22].

Conventional radiographs of the joint are limited, and interpretation of these exams is difficult [5, 7, 17, 23]. The bone changes of TMJ are best showed in CT images [3, 7, 23, 24].

OA is a chronic disease, and so, as all chronic process, shows destructive and reparative features, both many times occurring simultaneously. As previously described, this case showed on TC scan erosion areas, rough condylar surfaces, sclerosis areas, and bone outgrowths (osteophytes). According some authors, erosion and rough condylar surfaces with the loss of contour reflect the destructive stage of the disease, whilst sclerosis areas and bone outgrowths would be related to tissue repair [17].

Specific changes in the architecture of the subchondral trabecular bone due to accelerated bone turnover can form subchondral cysts called pseudocysts or Ely's cysts [1], which corroborated with the findings presented in our case. In symptom-free individuals, radiographic evidence of OA of the TMJ occurs in 14% to 44%. However, clinical evidence of the disease occurs in only 8% to 16% of the population [3]. In accordance with previous studies [25], the clinical symptoms in the present case were not consistent with the CT findings that showed the disease in a late stage. It reveals that, in some patients, degenerative lesions can be present with few or without symptoms and they can only be visibly detected by CT scan [23, 24].

It is accepted that OA and internal derangement (ID) may coexist in about one-third of the cases [26]. ID is considered the most common cause of severe TMJ pain and dysfunction. de Leeuw et al. (1996) found a significant correlation between disc position and the severity of degenerative changes of TMJ in radiographs in symptomatic and asymptomatic TMJ [27]. The best way to assess changes of the articular disc, condyle, and the articular eminence is by magnetic resonance imaging (MRI) of the TMJ [28–32]. In this case, we did not evaluate our patient's disc position, but the diagnosis of TMJ OA is doubtlessly based on clinical findings. No radiographic criterion is pathognomonic for rheumatoid diseases. All of them can show erosion, sclerosis, osteophytes, flattening, subchondral cysts, and a reduced joint space. However, reduced joint space, flattening of the condyle, and osteophytes have been reported to be more common in OA, whereas erosions in the condyle are more frequently found in RA [20, 33].

There are in the literature different types of treatment for TMJ OA, but in general, they fall into two lines: nonsurgical and surgical procedures. The treatment may be initially performed using conservative therapies, being surgery reserved for those cases where nonsurgical approach was not effective, and pain and the loss of function were resistant to conservative measures [26, 34].

Based on the aspects discussed, we concluded that, for the correct differential diagnosis of TMJ OA, it is necessary to unite medical history, physical examination, laboratory tests, and image findings. For image study, CT scan is considered the main imaging modality for assessing the osseous components of the TMJ OA.

## Figures and Tables

**Table 1:** Differential diagnosis among osteoarthritis (OA), rheumatoid arthritis (RA), and pain dysfunction syndrome (PDS) [3, 12, 13].

Findings	OA	RA	PDS
Pain	Localized	Diffuse	Irradiated
TMJ involvement	Symmetric or not	Symmetric	Symmetric or not
Subcutaneous nodes	Absent	Present (20%)	Absent
Type of hand swelling	Hard	Soft	Absent
Extra-articular findings	Absent	May be present	Absent
Morning stiffness	Absent	Present	Absent
Crepitation	Present	Rarely	Rarely
Clicking	Rarely	Absent	Present
Rheumatoid factor	Rarely present	Present	Absent
Erythrocyte sedimentation rate	Normal	Usually elevated	Normal
Synovial fluid	Normal	Inflammation	Normal
Radiographic findings	Erosive + exophytic (asymmetric cartilage loss)	Erosive (symmetric cartilage loss)	May be present
